# Pegivirus Infection in Domestic Pigs, Germany

**DOI:** 10.3201/eid2207.160024

**Published:** 2016-07

**Authors:** Christine Baechlein, Adam Grundhoff, Nicole Fischer, Malik Alawi, Doris Hoeltig, Karl-Heinz Waldmann, Paul Becher

**Affiliations:** University of Veterinary Medicine Hannover, Hannover, Germany (C. Baechlein, D. Hoeltig, K.-H. Waldmann, P. Becher);; German Center for Infection Research Partner Site Hannover–Braunschweig, Hannover (C. Baechlein, P. Becher);; German Center for Infection Research Partner Site Hamburg–Lübeck–Borstel, Hamburg (N. Fischer, A. Grundhoff);; Heinrich Pette Institute, Hamburg, Germany (A. Grundhoff, M. Alawi);; University Medical Center Hamburg–Eppendorf, Hamburg (N. Fischer, M. Alawi)

**Keywords:** pegivirus, viruses, Flaviviridae, infection, domestic pigs, high-throughput sequencing, RT-PCR, Germany

**To the Editor:** The family *Flaviviridae* includes many human and animal virus pathogens. Recently, in addition to the genera *Flavivirus*, *Hepacivirus*, and *Pestivirus*, a fourth genus, *Pegivirus*, has been identified (*1*). In addition to human pegiviruses, a range of phylogenetic, highly divergent pegiviral sequences have been identified in various animal species, including primates, bats, rodents, and horses (*2*). We report the detection of a porcine pegivirus (PPgV) in serum samples from pigs.

Initially, we investigated pooled serum samples by using high-throughput sequencing methods and isolated RNA from individual porcine serum samples by using the QIAmp Viral RNA Mini Kit (QIAGEN, Hilden, Germany). We prepared libraries compatible with Illumina (San Diego, CA, USA) sequencing from pooled samples and individual serum samples by using the ScriptSeq version 2 RNA-Seq Library Preparation Kit (Epicenter, Madison, WI, USA) and sequenced them by using a HiSeq 2500 (2 × 150 cycles paired-end; Illumina) for pooled samples and MiSeq (2 × 250 cycles paired-end; Illumina) for individual samples (*3*).

We conducted quantitative reverse transcription PCR (RT-PCR) by using a Quantitect-SYBR Green Assay (QIAGEN) and primers PPgV_fwd: 5′-CTGTCTATGCTGGTCACGGA-3′ and PPgV_rev: 5′-GCCATAGAACGGGAAGTCGC-3′. By using high-throughput sequencing of the pooled serum sample library (23,167,090 reads), we identified 1 contig (4,582 bp) that had distant nucleotide sequence simi-larity to bat pegivirus (69% and 4% sequence coverage) and 2 contigs (2,683 bp and 665 bp) that had 73% sequence coverage, thereby covering 8% and 37% of the identified sequence. RT-PCR with primers designed on basis of recovered sequences identified the sample containing pegivirus sequences. Subsequent MiSeq analysis (7,085,595 reads) of an RNA library prepared from a sample from 1 animal identified 1 contig (9,145 nt) with sequence similarity to pe-givirus sequences.

We performed 3′ end completion of the viral genome by rapid amplification of cDNA ends and identified the entire open reading frame of PPgV_903 encoding 2,972 aa (GenBank accession no. KU351669). Analysis of the pegivirus 5′-untranslated region identified a highly structured internal ribosome entry site motif (Technical Appendix), which was similar in structure to previously described 5′ untranslated region structures of other pegiviruses (*4**,**5*).

Pegiviruses do not encode a protein homologous to the capsid protein of other viruses of the family *Flaviviridae*, another common feature of pegiviruses (*6*). The presence of cleavage sites for cellular signal peptidases and viral proteases indicates that, similar to polyproteins of other pegiviruses and members of the genus *Hepacivirus*, the pegivirus polyprotein NH_2_-E1- E2-Px-NS2-NS3-NS4A-NS4B-NS5A-NS5B-COOH (E [envelope], NS [nonstructural], and Px [protein X]) is cleaved co-translationally and posttranslationally.

We tested 3 additional animals from the same breeding cohort for virus RNA at irregular intervals for 22 months. One animal was positive for pegivirus RNA for 7 months, and the other 2 animals had pegivirus RNA in serum for 16 and 22 months. None of these animals showed obvious clinical signs attributable to virus infection. Follow-up investigation of 455 serum samples from 37 swine holdings from Germany identified 10 (2.2%) samples from 6 pig holdings that contained pegivirus RNA. We obtained 2 additional near full-length genomic sequences (PPgV_80F and PPgV_S8-7) from 2 animals in different herds by high-throughput sequencing, RT-PCR, and Sanger sequencing (GenBank accession nos. KU351670 and KU351671).

Phylogenetic analyses of complete coding regions showed the close relationship of the 3 pegivirus sequences from Germany. These 3 sequences formed a separate clade within the genus *Pegivirus* (Figure). Pairwise comparison between PPgV_903 and the other 2 pegivirus sequences showed strong nucleotide identities (96.0%–98.4%). A distance scan over the entire polyprotein showed genetic distance to other pegiviruses and demonstrated that NS3 and NS5B contain the most conserved regions among pegivirus polyproteins (Technical Appendix).

**Figure:**
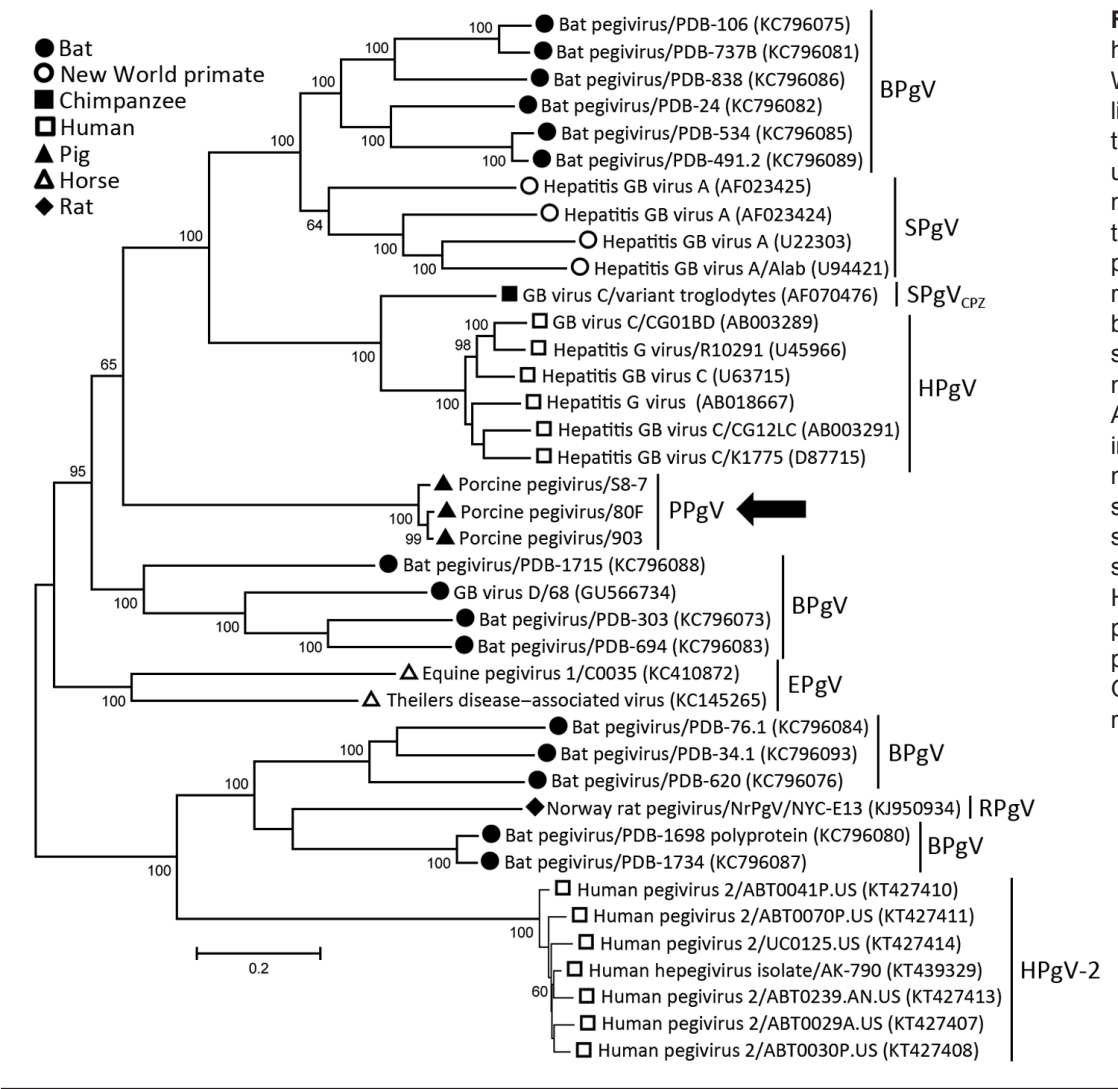
Phylogenetic analysis of human and animal pegiviruses. We constructed a maximum-likelihood tree on the basis of the complete coding region and used the general time reversible model for modeling of substitutions. Bootstrap analysis was performed with 200 replicates. Numbers along branches are percentage bootstrap values. GenBank accession numbers are in parentheses. Arrow indicates viruses isolated in this study. Scale bar indicates nucleotide substitutions per site. BPgV, bat pegivirus, SPgV, simian pegivirus; SPgV_CPZ_, simian pegivirus (chimpanzee); HPgV, human pegivirus; PPgV, porcine pegivirus; EPgV, equine pegivirus; RPgV, rodent pegivirus. GB viruses have recently been reclassified as pegiviruses.

In horses, 2 distinct pegiviruses that had different potentials to cause clinical disease in infected animals have been described (*4**,**7*). No obvious clinical effects were observed in pegivirus-infected animals during our study. However, potential consequences of viral infection for animal health and food production need to be explored more closely under field and experimental conditions. Pegiviruses can interact with the immune system of the host. Co-infection with human pegivirus and HIV can have beneficial effects, which result in decreased retroviral loads and delayed disease progression (*8*).

It will be useful to investigate whether co-infections with pegiviruses can influence clinical manifestations of infectious diseases of swine, including multifactorial diseases such as postweaning multisystemic wasting syndrome, in which unknown immune modulating virus infections have been suggested to influence the degree of clinical illness (*9*). RNA viruses have considerable potential to adapt to new environmental conditions and to overcome host restrictions (*10*). Until now, the host tropism of PPgV has not been investigated in detail. Therefore, additional studies will be required to elucidate whether the spectrum of potential hosts might include other farm or companion animals, and whether the virus might be able to infect humans.

Technical Appendix Additional information on pegivirus infection in domestic pigs, Germany.

## References

[1] Stapleton JT, Bukh J, Muerhoff AS, Foung S, Simmonds P. Assignment of human, simian and bat pegiviruses (previously described as GBV-A, GBV-C, and GBV-D) as members of a new genus (*Pegivirus*) within the *Flaviviridae* [cited 2015 Oct 21]. http://www.ictvonline.org/proposals/2012.011a-dV.A.v2.Pegivirus.pdf

[2] Thézé J, Lowes S, Parker J, Pybus OG. Evolutionary and phylogenetic analysis of the hepaciviruses and pegiviruses. Genome Biol Evol. 2015;7:2996–3008 . 10.1093/gbe/evv202 26494702 PMC5635594

[3] Baechlein C, Fischer N, Grundhoff A, Alawi M, Indenbirken D, Postel A, Identification of a novel hepacivirus in domestic cattle from Germany. J Virol. 2015;89:7007–15. 10.1128/JVI.00534-15 25926652 PMC4473572

[4] Kapoor A, Simmonds P, Cullen JM, Scheel TK, Medina JL, Giannitti F, Identification of a pegivirus (GB virus-like virus) that infects horses. J Virol. 2013;87:7185–90. 10.1128/JVI.00324-13 23596285 PMC3676142

[5] Simons JN, Desai SM, Schultz DE, Lemon SM, Mushahwar IK. Translation initiation in GB viruses A and C: evidence for internal ribosome entry and implications for genome organization. J Virol. 1996;70:6126–35. 8709237 10.1128/jvi.70.9.6126-6135.1996 PMC190635

[6] Stapleton JT, Foung S, Muerhoff AS, Bukh J, Simmonds P. The GB viruses: a review and proposed classification of GBV-A, GBV-C (HGV), and GBV-D in genus *Pegivirus* within the family *Flaviviridae.* J Gen Virol. 2011;92:233–46 . 10.1099/vir.0.027490-0 21084497 PMC3081076

[7] Chandriani S, Skewes-Cox P, Zhong W, Ganem DE, Divers TJ, Blaricum AJ, Identification of a previously undescribed divergent virus from the *Flaviviridae* family in an outbreak of equine serum hepatitis. Proc Natl Acad Sci U S A. 2013;110:E1407–15. 10.1073/pnas.1219217110 23509292 PMC3625295

[8] Schwarze-Zander C, Blackard JT, Rockstroh JK. Role of GB virus C in modulating HIV disease. Expert Rev Anti Infect Ther. 2012;10:563–72 . 10.1586/eri.12.37 22702320 PMC3499065

[9] Grau-Roma L, Fraile L, Segalés J. Recent advances in the epidemiology, diagnosis and control of diseases caused by porcine circovirus type 2. Vet J. 2011;187:23–32. 10.1016/j.tvjl.2010.01.018 20211570

[10] Rosenberg R. Detecting the emergence of novel, zoonotic viruses pathogenic to humans. Cell Mol Life Sci. 2015;72:1115–25. 10.1007/s00018-014-1785-y 25416679 PMC4629502

