# Supplementary material for: Pegivirus Infection in Domestic Pigs, Germany
Source: Emerg Infect Dis. 2016 Jul;22(7):1312–4. doi: 10.3201/eid2207.160024 (PMC4918184; doi:10.3201/eid2207.160024)
Supplement: Technical Appendix — Additional information on pegivirus infection in domestic pigs, Germany. [file 16-0024-Techapp-s1.pdf]

Article DOI: http://dx.doi.org/10.32021/eid2207.160024

Pegivirus Infection in Domestic Pigs, Germany

Technical Appendix

We used Trimmomatic (*1*) to remove adaptor sequences and trim low-quality bases (phred quality score <15) from the 3′-end of the reads. The reads were then aligned to the assembled consensus sequence of porcine pegivirus PPgV_903 with the Burrows Wheeler Aligner (bwa mem) (*2*). Samtools (*3*) was used for format conversion, sorting, and removal of putative PCR duplicates. Variants were called with FreeBayes (*4*). The minimal fraction of observations required to call an alternate allele was reduced to 5%, whereas the minimum absolute number of required observations was increased to 5. To increase stringency, FreeBayes' standard filters were used and results were filtered to exclude variants with a quality score <21.

References

1. Bolger AM, Lohse M, Usadel B. Trimmomatic: a flexible trimmer for Illumina sequence data. Bioinformatics. 2014;30:2114–20. PubMed http://dx.doi.org/10.1093/bioinformatics/btu170

2. Li H, Durbin R. Fast and accurate short read alignment with Burrows-Wheeler transform. Bioinformatics. 2009;25:1754–60. PubMed http://dx.doi.org/10.1093/bioinformatics/btp324

3. Li H, Handsaker B, Wysoker A, Fennell T, Ruan J, Homer N, et al. The sequence alignment/map format and SAMtools. Bioinformatics. 2009;25:2078–9. PubMed http://dx.doi.org/10.1093/bioinformatics/btp352

4. Garrison E, Marth G. Haplotype-based variant detection from short-read sequencing. ArXiv12073907 Q-Biol, 2012 [cited 2016 Jan 5]. http://arxiv.org/abs/1207.3907

Table. Variant calling for genomes of pegiviruses*

Position	PPgV_903 reference†	Variant sequence‡	Variant frequency PPgV_903§	Variant frequency PPgV_80F§
321	C	T	0	1
330	T	A	0.36	0
559	A	G	0	0.97
704	T	C	0	1
755	C	T	0	1
767	C	T	0	1
795	A	T	0	1
858	T	C	0	1
867	G	T	0.62	0
869	C	T	0	1
914	A	G	0.71	0
980–983	CAGC	TAGT	0	1
1098	A	G	0	1

Position	PPgV_903 reference†	Variant sequence‡	Variant frequency PPgV_903§	Variant frequency PPgV_80F§
1170	C	T	0.03	1
1181	T	C	0	0.67
1196–1199	TCTG	CCTA	0	0.95
1226	C	T	0.06	1
1265	T	C	0	1
1271	C	T	0	1
1455	T	C	0	1
1465	T	A	0.17	0
1469	T	C	0	1
1511	C	T	0	1
1522	T	C	0.44	0
1539	C	T	0	1
1574	C	T	0	1
1586	T	C	0	1
1646	T	C	0.16	0
1718	C	T	0	1
1727	T	C	0	1
1832	C	T	0	1
1895	T	C	0	1
1901	C	T	0	1
1907	T	C	0.02	1
2057	A	G	0	1
2066	G	A	0	0.98
2096	C	T	0	1
2102	C	T	0	1
2195	C	T	0.07	1
2231	T	C	0	1
2300	T	C	0	1
2375	C	T	0	1
2545	A	G	0	1
2634	C	T	0	0.87
2672	C	T	0	1
2732	C	T	0	1
2741	T	C	0.03	0.96
2837	T	C	0	1
2891	T	C	0	1
3173	T	C	0.12	0.02
3182	C	T	0	1
3260–3262	CAA	TAG	0	1
3378	T	C	0	1
3467	C	T	0.03	1
3575	T	C	0	0.84
3587	C	T	0.38	0
3614	G	A	0	1
3764	G	A	0	1
3911	T	C	0	1
3917	A	T	0.36	0
4001	T	C	0	1
4013	T	C	0	1
4097	C	T	0.19	0
4160	C	T	0	1
4181	A	G	0.11	1
4196	T	C	0	1
4211	G	A	0	0.9
4346	C	T	0	0.48
4424	C	T	0	1
4478	C	T	0	1
4541	A	G	0	0.99
4700	A	G	0	0.38
4784	G	A	0	1
4817	C	T	0.26	0
4835	T	C	0	0.98
4940	A	G	0	1
4985	T	C	0	1
5114	C	T	0.07	1
5192	T	C	0	1
5441	T	C	0	0.98
5474	A	G	0	1
5492	T	C	0.44	1
5732	T	C	0	1
5936	C	T	0	1
5999	G	A	0	1
6074–6077	CGTT	TGTC	0	1
6128	A	G	0	1
6191	C	T	0	1

Position	PPgV_903 reference†	Variant sequence‡	Variant frequency PPgV_903§	Variant frequency PPgV_80F§
6220	G	A	0	0.82
6297	C	T	0	1
6323	C	T	0	1
6338	T	C	0.05	1
6362	T	C	0.33	1
6389	T	C	0	1
6449	C	G	0	1
6482	T	C	0	1
6509	C	T	0	1
6542	A	G	0	1
6554	T	C	0	1
6632	T	C	0.38	0
6722	T	C	0	1
6744	T	C	0	1
6761–6764	TTTG	CTTA	0	1
6770	G	T	0	1
6803–6807	CGTGA	TGTGG	0	1
6824	C	T	0.15	0
6878	T	C	0	1
6892	T	C	0.53	1
6926	A	G	0.01	0.98
6965	T	C	0.01	0.89
7088	A	G	0	1
7343	G	A	0	0.9
7349	T	C	0	1
7364	T	C	0	1
7460–7466	AGCGACG	GGCAACA	0	1
7493	C	T	0	1
7517	T	C	0	0.92
7601	T	C	0.05	1
7662	C	T	0.04	1
7694	T	C	0	1
7745	T	C	0	1
7751	A	G	0.25	0
7763	A	C	0	1
7931	T	C	0.05	0.87
7967	A	G	0	0.95
7997	C	T	0.13	0
8036	G	A	0.01	1
8129	A	G	0	1
8207	T	C	0	1
8309	C	T	0.24	0.04
8330	T	C	0	0.98
8351	C	T	0.16	0
8384	G	A	0	1
8435	A	G	0	1
8522	C	T	0	1
8537	G	A	0	1
8544	G	A	0	1
8549	A	G	0	1
8566	G	A	0	1
8573	T	C	0.06	1
8591	C	T	0	1
8600	C	T	0.03	1
8630	A	C	0	1
8639	A	G	0	1
8666	C	T	0.28	0.02
8708	C	T	0.03	1
8738	T	C	0.12	0.91
8747	T	C	0	1
8789	T	C	0	1
8828	T	C	0	0.67
8930	T	A	0	1
9032	C	T	0.02	1
9050	T	C	0	1
9077	C	T	0.06	1

*PPgV, porcine pegivirus.
†Reference nucleotide sequence as assembled from PPgV_903 sequencing reads. The sequence served as a common reference for the analysis of sequence variants in PPgV_80F- and PPgV_903-infected animals.
‡Variant nucleotide sequence as observed in PPgV_80F- or PPgV_903-infected animals.
§Frequency of variant nucleotide sequences in PPgV_80F or PPgV_903, respectively (0, 0%; 1, 100%).

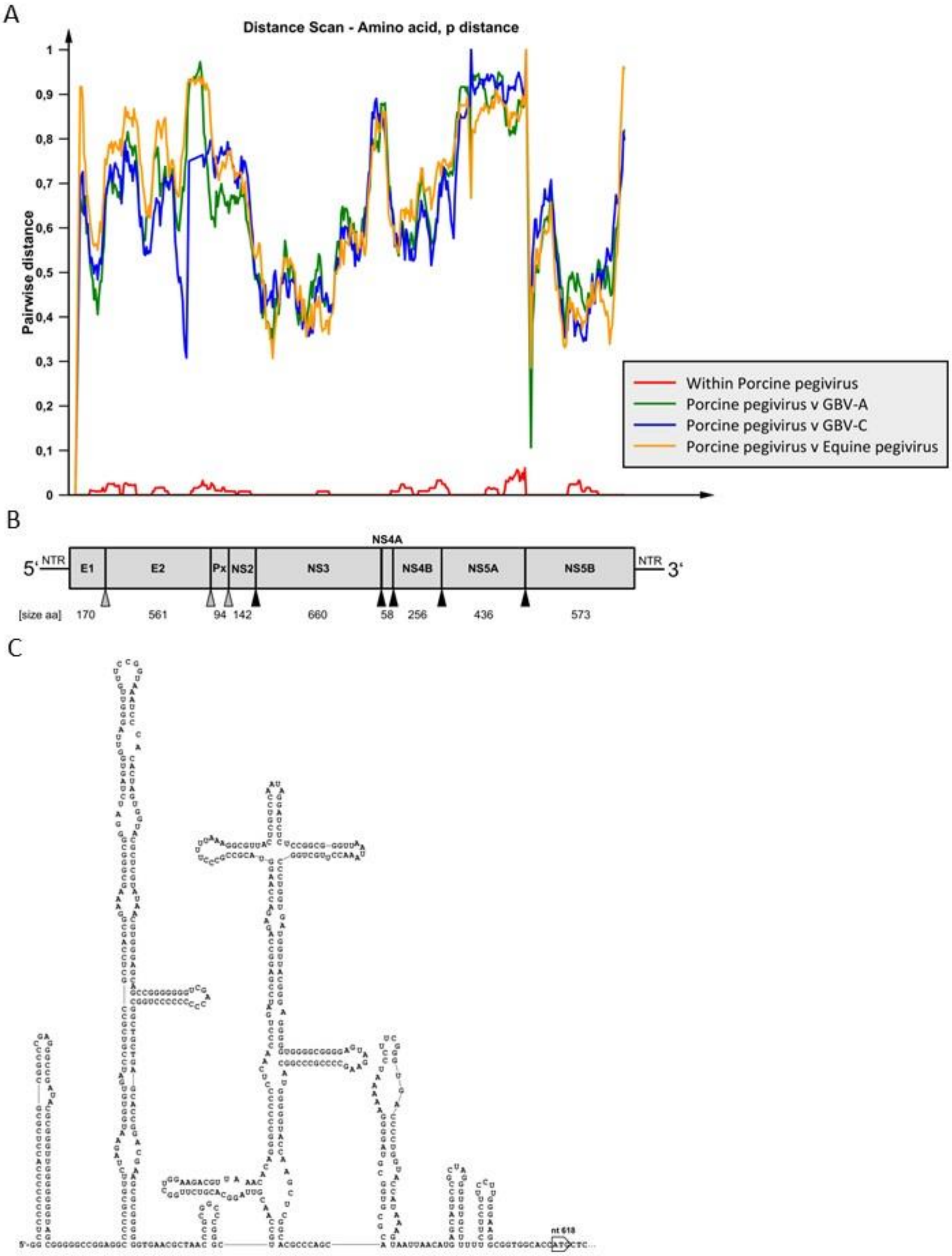

Technical Appendix Figure. A) Pairwise amino acid distances plotted over the complete polyprotein of pegiviruses. A schematic drawing of the pegivirus polyprotein is depicted below indicating corresponding genomic regions. GBV, hepatitis G virus. B) Schematic drawing of the pegivirus polyprotein. Putative cleavage sites specific for cellular signal peptidases are indicated in gray, and cleavage sites typical for viral proteases are indicated in black. The length of each putative mature viral protein is given below. E1, E2, envelope glycoproteins; P, p protein; NS, nonstructural proteins.

C) Schematic drawing of the pegivirus internal ribosome entry site structure within the 5′-nontranslated region (NTR). RNA folding was performed by using mfold (http://unafold.rna.albany.edu/?q=mfold/rna-folding-form) (default settings). The box indicates the start codon of the polyprotein.
